# The Implementation of an Electronic Medical Record in a German Hospital and the Change in Completeness of Documentation: Longitudinal Document Analysis

**DOI:** 10.2196/47761

**Published:** 2024-01-19

**Authors:** Florian Wurster, Marina Beckmann, Natalia Cecon-Stabel, Kerstin Dittmer, Till Jes Hansen, Julia Jaschke, Juliane Köberlein-Neu, Mi-Ran Okumu, Carsten Rusniok, Holger Pfaff, Ute Karbach

**Affiliations:** 1 Chair of Quality Development and Evaluation in Rehabilitation, Institute of Medical Sociology, Health Services Research, and Rehabilitation Science, Faculty of Human Sciences & Faculty of Medicine and University Hospital Cologne, University of Cologne Cologne Germany; 2 Center for Health Economics and Health Services Research, University of Wuppertal Wuppertal Germany

**Keywords:** clinical documentation, digital transformation, document analysis, electronic medical record, EMR, Germany, health services research, hospital, implementation

## Abstract

**Background:**

Electronic medical records (EMR) are considered a key component of the health care system’s digital transformation. The implementation of an EMR promises various improvements, for example, in the availability of information, coordination of care, or patient safety, and is required for big data analytics. To ensure those possibilities, the included documentation must be of high quality. In this matter, the most frequently described dimension of data quality is the completeness of documentation. In this regard, little is known about how and why the completeness of documentation might change after the implementation of an EMR.

**Objective:**

This study aims to compare the completeness of documentation in paper-based medical records and EMRs and to discuss the possible impact of an EMR on the completeness of documentation.

**Methods:**

A retrospective document analysis was conducted, comparing the completeness of paper-based medical records and EMRs. Data were collected before and after the implementation of an EMR on an orthopaedical ward in a German academic teaching hospital. The anonymized records represent all treated patients for a 3-week period each. Unpaired, 2-tailed *t* tests, chi-square tests, and relative risks were calculated to analyze and compare the mean completeness of the 2 record types in general and of 10 specific items in detail (blood pressure, body temperature, diagnosis, diet, excretions, height, pain, pulse, reanimation status, and weight). For this purpose, each of the 10 items received a dichotomous score of 1 if it was documented on the first day of patient care on the ward; otherwise, it was scored as 0.

**Results:**

The analysis consisted of 180 medical records. The average completeness was 6.25 (SD 2.15) out of 10 in the paper-based medical record, significantly rising to an average of 7.13 (SD 2.01) in the EMR (t_178_=–2.469; *P*=.01; *d*=–0.428). When looking at the significant changes of the 10 items in detail, the documentation of diet (*P*<.001), height (*P*<.001), and weight (*P*<.001) was more complete in the EMR, while the documentation of diagnosis (*P*<.001), excretions (*P*=.02), and pain (*P*=.008) was less complete in the EMR. The completeness remained unchanged for the documentation of pulse (*P*=.28), blood pressure (*P*=.47), body temperature (*P*=.497), and reanimation status (*P*=.73).

**Conclusions:**

Implementing EMRs can influence the completeness of documentation, with a possible change in both increased and decreased completeness. However, the mechanisms that determine those changes are often neglected. There are mechanisms that might facilitate an improved completeness of documentation and could decrease or increase the staff’s burden caused by documentation tasks. Research is needed to take advantage of these mechanisms and use them for mutual profit in the interests of all stakeholders.

**Trial Registration:**

German Clinical Trials Register DRKS00023343; https://drks.de/search/de/trial/DRKS00023343

## Introduction

The digital transformation of the health care system is considered an essential subject to meet current and future societal challenges such as an aging population or rising health care expenditures while at the same time maintaining a high quality of care [1]. An important early step in hospitals’ digitalization and a fundamental requirement for expanding digital maturity is the implementation of an electronic medical record (EMR) [2]. This EMR is considered to be an **“**electronic record of health care information of an individual that is created, gathered, managed, and consulted by authorized clinicians and staff within 1 health care organization” [3] and replaces the internal clinical documentation on preprinted paper-based charts. Studies show that the implementation of an EMR can lead to various improvements in the clinical context (eg, in the availability of information [4], coordination of care [5], or patient safety [6]). Moreover, the EMR facilitates the secondary usage of the documented data for research purposes through its digital accessibility [7]. To reach those benefits, it is indispensable that the EMR contain documentation that is of high quality. However, there are varying definitions regarding the quality of documentation. In that matter, the Institute of Medicine defined completeness, legibility, accuracy, and meaning as the main aspects of a medical record’s data quality [8]. For those, the completeness of documentation was shown to be the most common dimension of data quality when empirically analyzing the documentation in EMRs [9], and it was highlighted to be especially important for secondary uses such as big data analyses [10].

Our recent systematic review also stated the completeness of documentation as the state of the art for the comparison of paper-based and EMRs [11]. This comparison is important since the implementation of an EMR and the associated transition from handwritten documentation to digital documentation can heavily affect the documentation subject since the transition offers the possibility to adjust which information has to be documented in which way [12]. For example, digitization enables the adoption of certain functionalities that can alter the completeness of documentation, like automatically transferring information from other digital devices to the EMR [13]. Moreover, when working with the EMR, information can be documented remotely, while the paper-based medical record had to be located and physically accessed first. In this matter, several studies conducted in the inpatient setting showed increased completeness in the EMR compared to the paper-based medical record, for example, for the documentation of signs and symptoms [13,14], weight and height, or malnutrition screening [15]. This suggests that the implementation of an EMR might lead to improvements in the completeness of documentation in general. It is therefore the main purpose of this study to evaluate the change in completeness due to the implementation of an EMR in an inpatient setting. Literature already provides proof of a change of completeness in regard to some specific documented information that is analyzed in this work (eg, the documentation of vital signs) [13,14]. Those empirical results might thus be validated for the presented work’s specific setting and discipline. In addition, some of the information that is analyzed in this work is not described in literature yet (eg, the documentation of pain). It is examined for the first time with regard to changes in completeness after the implementation of an EMR.

The knowledge gained can not only support the implementation of new EMRs but could also help understand and optimize arising changes in documentation when existing EMRs need to be adapted [16,17]. This is an important aspect, as the implementation of new EMRs is described as one of the most important interventions to improve the quality of documentation [18]. In this process, mechanisms affecting the completeness of documentation in medical records are not completely understood [10]. On the other hand, this knowledge is needed to fulfill reported educational needs regarding how to reach the optimum quality of documentation [19]. In this context, this study contributes to a more comprehensive understanding of the impact of an EMR on the quality of documentation.

## Methods

### Overview

This study follows the “Strengthening the Reporting of Observational Studies in Epidemiology” (STROBE) statement [20] whenever it is applicable. It offers reporting standards to ensure the reporting of any important information in empirical research studies. A checklist with details, where the STROBE information is mentioned in the manuscript, can be found in Multimedia Appendix 1.

### Ethical Considerations

The study has been approved by the ethics committee of the Medical Faculty of the University of Cologne, Germany (20-1349). All data was anonymized at all times during the scientific analysis. No compensation was paid.

### Setting and Participants

**Figure 1:**
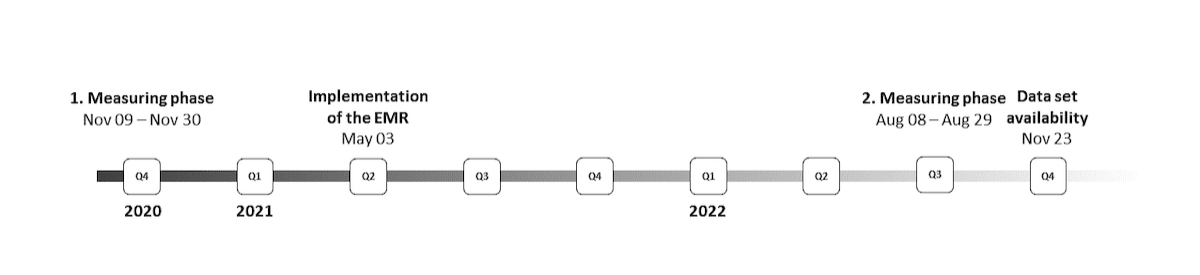
Data collection. EMR: electronic medical record.

The study took place as part of the research project eCoCo, which Beckmann et al [21] described in detail. Within the eCoCo project, the researchers collected various types of data (observations, surveys, interviews, documents, and administrative data) to investigate a possible change in interprofessional collaboration and clinical workflows following the implementation of an inpatient EMR. This study is part of the related work package on documentation content and quality, which took place in a large academic teaching hospital in Germany. The hospital replaces its internal documentation on preprinted paper-based charts with a commercial EMR system (Meona; Mesalvo Freiburg GmbH). The EMR runs on multiple computers that can be moved flexibly over the ward on trolleys. The study follows a pre-post design, retrospectively analyzing the content of the medical records before and after the implementation of the EMR on the hospitals’ orthopaedical ward. Within the first measuring phase, the paper-based medical records were provided as a digital copy of the paper sheets. Those paper-based records represent all patients who were treated on the ward during the last 3 weeks in November 2020 (t0). After 6 months, employees received training on how to use the EMR before the implementation of the EMR took place in May 2021. The EMRs were again provided as a digital copy within a second measuring phase, representing all patients who were treated on the same ward during the first 3 weeks of August 2022 (t1). This resulted in a gap of 15 months between the first and second measuring phases. The complete data set was available to the research team in November 2022 (Figure 1). The hospital provided anonymized medical records to the research team after the records were archived and cleared of sensitive personal data (eg, the patient’s name or date of birth) in the hospital’s internal processes. Any assignment of the patient data or linking of the records’ contents to any individual patient was therefore impossible for the research team, which is, thus, in compliance with the European Union General Data Protection Regulation. This also implies the absence of sociodemographic information for describing the compared samples. The hospital’s mandatory annual quality report, which is available to the public through a designated database [22], is therefore used to describe the ward’s patient sample and the performed treatments in general. This allows an approximation to a description and comparison of the compared samples in terms of their *International Classification of Diseases* (*ICD*)–diagnoses distribution.

### Study Objective

**Figure 2:**
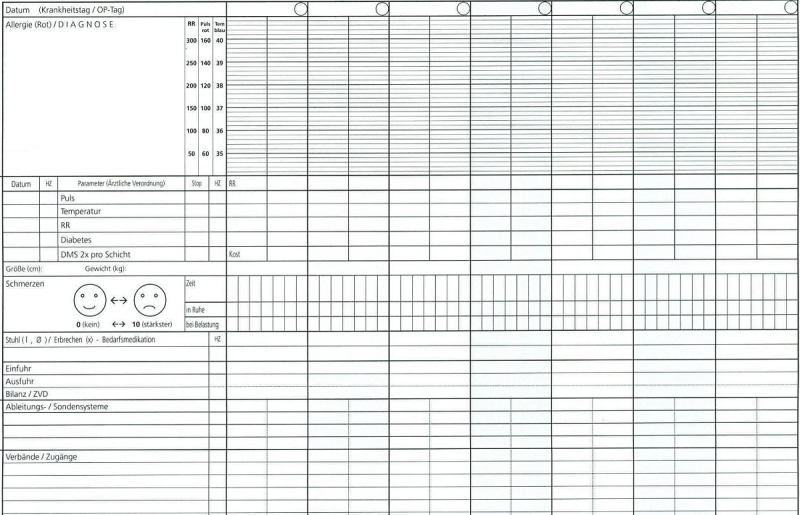
Paper-based fever chart.

**Figure 3:**
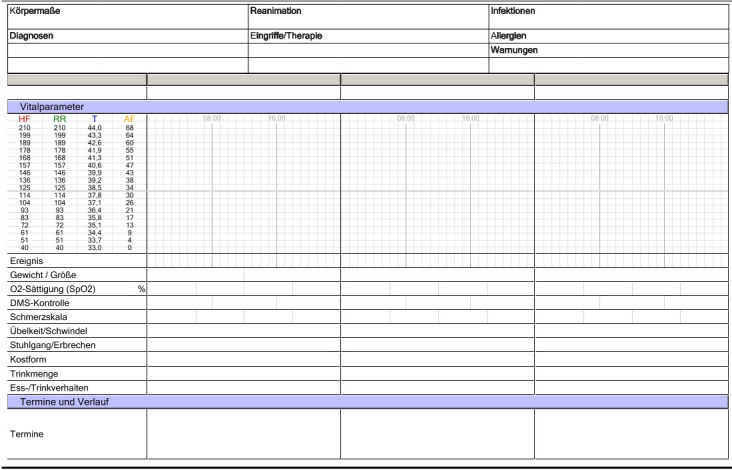
Electronic fever chart.

To answer the question of a possible change in completeness, the records were analyzed by content [23]. The change from paper-based documentation to EMRs always offers the opportunity to fundamentally change the structure of the records. This was shown exemplarily by Montagna et al [12] when the documentation as a continuously written text in the paper-based record was changed to a list of events in the EMR. It is therefore important to ensure the comparability between the 2 record types for the purpose of analyzing a possible change in their completeness. To achieve comparability, the medical records progress note was selected as a specific object of interest for this study’s analyses since it retained the same structure and format in both record types. Part of this progress note is the fever chart (Figures 2 and 3), which includes basic details about vital signs, personal health data, etc [24].

All information that was commonly documented in both of the 2 record types (paper-based and electronic) became part of this work. Weiskopf and Weng [9] described this selection mechanism for assessing data quality based on the parallels between the EMR and the paper-based record. This procedure resulted in a total of 10 key items that were analyzed for completeness in this work: blood pressure, body temperature, diagnosis, diet, excretions, height, pain, pulse, reanimation status, and weight. The documentation of this information is equally possible and performed by nurses and physicians. However, there is no information available about who specifically entered the information.

All of those items should be documented immediately when patient care begins on the ward [25]. However, while the documentation of vital signs can take place up to several times a day, the documentation of the patient’s diet usually occurs once a day, and the documentation of the reanimation status (patient’s preference regarding a possible resuscitation) is probably documented only once per hospital stay. Because of these varying documentation practices and to ensure comparability, the analysis focuses on certain documentation in the progress notes that was entered on the first day of patient care on the ward. With regard to the documentation of a diagnosis, it is therefore the diagnosis with which a patient is admitted to the hospital. This diagnosis is mainly responsible for the allocation to specific medical specialties as well as a certain ward and does not necessarily have to match the final diagnosis at the time of discharge, which is important for reimbursement purposes.

### Statistical Analysis of Completeness

For every record, each of the 10 items received a dichotomous score of 1 if it was documented on the first day of patient care on the ward; otherwise, it was scored as 0. This resulted in a percentage of completeness for each item per record type. Chi-square tests for independence were used to assess statistically significant differences in the percentage of completeness per item between the 2 record types. Relative risks were calculated for the association between the electronic record type and a possible increase in completeness. To improve the reliability of the associated confidence intervals, they were calculated with 5000 bootstrap replications since the original sample sizes are unbalanced. Moreover, the overall completeness was assessed as sum of the 10 items, resulting in a mean score of completeness per record type ranging from 0 (no item documented) to 10 (all 10 items documented). Those mean scores of completeness per record type were analyzed for equality of variance and statistical difference using unpaired, 2-tailed *t* tests. Assumptions were checked using several methods (normal distribution: QQ plots and Shapiro-Wilk test; homogeneity of variances: Levene test; and linearity: scatter plot). The level of significance was set to be *P*<.05 for all calculations. The data were stored in Microsoft Excel (Microsoft Corp) and analyzed in December 2022 using SPSS software (version 29; IBM Corp).

## Results

### Participants

During the first measuring phase (November 2020), a total of 44 patients (paper-based) were treated on the orthopaedical ward. They were encountering a total of 136 treated patients (electronic) during the second measuring phase (August 2022). This resulted in a total of 180 medical records that became part of this analysis. Due to the data protection regulation and the accompanied anonymization of the records data, there is no information regarding the demographics of the specific study population. Therefore, the ward’s ICD-diagnosis distribution is given as an approximation of a sample description. In 2020, the 3 most frequently coded diagnoses for the orthopaedical ward were complications of internal orthopedic prosthetic devices, implants and grafts (ICD-T84), dorsalgia (ICD-M54), and fracture of shoulder and upper arm (ICD-S42). This report is not yet published for 2022, but the top 3 treated diagnoses in 2019 or 2021 were similar to those in 2020 (Table 1). It can therefore be expected that the treated diagnoses will be similar in 2022, too. Another supporting fact is that the most frequently performed procedure (surgical access to the lumbar spine, the sacrum, or the coccyx [coded as OPS-5-032 in the German adaptation of the International Classification of Procedures in Medicine which is part of the coding system for hospitals reimbursement]) was the same in all 3 years (2019-2021).

**Table 1:** Most frequently coded diagnoses.

ICD^a^ Code		Values, n^b^/N^c^ (%)
**2019**		
	Dorsalgia (ICD-M54)	213/3147 (6.77)
	Other spondylopathies (ICD-M48)	133/3147 (4.23)
	Fracture of forearm (ICD-S52)	131/3147 (4.16)
**2020**		
	Complications of internal orthopedic prosthetic devices, implants and grafts (ICD-T84)	166/2912 (5.7)
	Dorsalgia (ICD-M54)	148/2912 (5.08)
	Fracture of shoulder and upper arm (ICD-S42)	121/2912 (4.16)
**2021**		
	Complications of internal orthopedic prosthetic devices, implants and grafts (ICD-T84)	164/3091 (5.3)
	Dorsalgia (ICD-M54)	163/3091 (5.27)
	Fracture of forearm (ICD-S52)	159/3091 (5.14)

^a^ICD: International Classification of Diseases.

^b^Frequency of coded diagnosis.

^c^Total inpatient cases.

### Change of Completeness

**Figure 4:**
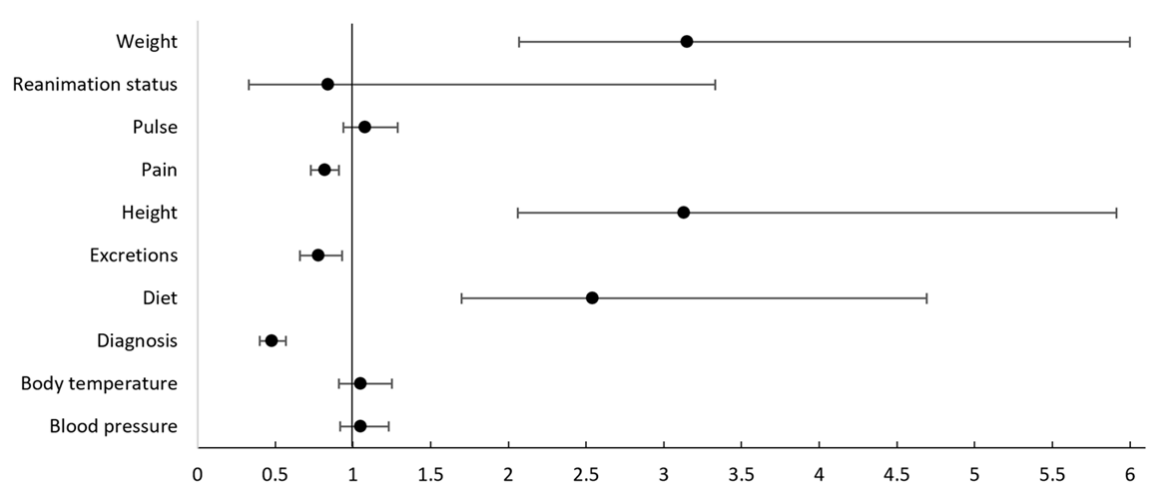
Forest plot of relative risks.

The mean number of documented items was 6.25 (SD 2.15) out of 10 in paper-based medical records and 7.13 (SD 2.01) out of 10 in EMRs. The Levene test confirmed the homogeneity of variances. The Shapiro-Wilk test did not confirm normal distributions, but the QQ plots show an approximation to a normal distribution and a comparable degree of normality (Multimedia Appendix 2). The unpaired *t* test confirmed the EMRs were statistically significantly more complete than the paper-based medical records under equal variances in the 2 record types (t_178_=–2.469; *P*=.01; *d*=–0.428). When looking at the 10 items separately, data from chi-square tests showed that the documentation of diet increased from being present in 30% (13/44) of the paper-based medical record to 75% (102/136; *P*<.001) in the EMR, height from 27% (12/44) to 85.3% (116/136; *P*<.001), and weight from 27% (12/44) to 86% (117/136; *P*<.001). At the same time, documentation of diagnosis decreased from being present in 100% (44/44) of the paper-based medical records to 49% (66/136; *P*<.001) in the EMR, excretions from 86% (38/44) to 68% (92/136; *P*=.02), and pain from 95% (42/44) to 78% (106/136; *P*=.008). The documentation of vital signs such as blood pressure (*P*=.47), body temperature (*P*=.497), and pulse (*P*=.28) remained unchanged on a high level of completeness, while the documentation of reanimation status (*P*=.73) remained unchanged on a low level of completeness (Table 2). Positive relative risks (Figure 4) illustrate the association of the electronic record type (exposure) with complete documentation (outcome). The confidence intervals represent 5000 bootstrap replications.

**Table 2:** Change of completeness.

Variable	Type of record		Chi-square (*df*)	*P* value	RR^a^ (95% CI)
	Paper (n=44), n (%)	Electronic (n=136), n (%)			
Blood pressure	37 (84)	120 (88.2)	0.5 (1)	.47	1.05 (0.92-1.23)
Body temperature	36 (81.8)	117 (86)	0.5 (1)	.497	1.05 (0.91-1.25)
Diagnosis	44 (100)	66 (48.5)	37.1 (1)	<.001	0.48 (0.40-0.57)
Diet	13 (29.6)	102 (75)	29.8 (1)	<.001	2.54 (1.70-4.69)
Excretions	38 (86.4)	92 (67.7)	5.8 (1)	.02	0.78 (0.66-0.93)
Height	12 (27.3)	116 (85.3)	54.5 (1)	<.001	3.13 (2.06-5.91)
Pain	42 (95.4)	106 (77.9)	7.0 (1)	.008	0.82 (0.73-0.91)
Pulse	36 (81.8)	120 (88.2)	1.2 (1)	.28	1.08 (0.94-1.29)
Reanimation status	5 (11.4)	13 (9.6)	0.1 (1)	.73	0.84 (0.33-3.33)
Weight	12 (27.3)	117 (86)	56.5 (1)	<.001	3.15 (2.07-6.00)

^a^RR: relative risk.

## Discussion

### Principal Findings and Comparison to Previous Work

The main findings of this study confirm an improved completeness of the analyzed information in the EMR on average. This provides further evidence for the suggestion that the general completeness of documentation can improve after the implementation of an EMR. The findings align with the results of similar studies, showing improvements in other data quality dimensions like the accuracy [26] or legibility [27] of documentation. However, when looking at the completeness of the analyzed 10 items in detail, the improvements can only be seen in 3 out of 10 items (diet, height, and weight), while 3 different items exhibited a deterioration in completeness (diagnosis, excretions, and pain). This links to the results of Coffey et al [28], who found 5 of their 11 analyzed items to be more complete while also proving 1 of their elements to be less complete. The reason for the variation in the change in completeness may lie in the mechanism of how information reaches the record. In the paper-based medical records, all information was documented by hand by the various professional groups. EMRs, on the other hand, offer technical features, for example, automatically obtaining information from other digital sources, like patients’ health insurance data [29]. This was manifested as a possible mechanism by Jang et al [30], who showed improved completeness in the EMR for the automatically filled information but not for the manually documented ones.

The analysis shows that roughly every second EMR was missing the documentation of a diagnosis. This is a remarkable change, as it was present in every paper-based record (44/44, 100% vs 66/136, 48.5%). In the first place, it must be clarified that the diagnosis is determined by a physician who enters it into an independently run hospital information system (HIS). This documented diagnosis can also be a preliminary diagnosis, which is used for distribution to the clinical disciplines and is present for every admitted patient. The HIS was already in operation when medical staff was still using the paper-based preprints for documentation purposes. After the EMR’s implementation, the HIS was still in operation along with the EMR. That being said, it is undisputed that during the paper-based period as well as the electronic period, a diagnosis was indeed present for the patients. In the paper-based period, the diagnosis was transferred manually from the HIS into the paper-based preprints, when a record for a recently admitted patient was prepared by a nurse. Since the HIS and the EMR are produced by different software developers, the diagnosis cannot be transferred automatically from the HIS into the EMR. Due to this noninteroperability of the 2 independent digital systems, the manual transfer is still necessary in the electronic period. With the drop of completeness in mind, this double documentation was accepted and carried out in the period of the paper-based record. In the electronic period, the described double documentation has decreased. One possible explanation is that the HIS was not automatically accessible, when an employee had the paper-based record at hand. With the introduction of the EMR, the availability of the EMR became synonymous with the availability of the HIS, since both are accessible from a computer. Therefore, the transfer of the diagnosis from the HIS to the EMR may no longer have been considered necessary. Nevertheless, the reason for this difference remaining unclear illustrates that the sole analyzation of completeness of the documentation alone does not provide sufficient information about the actual quality of the provided treatment. In that matter, it must also be highlighted that the record can contain additional qualitative data entries, like free texts, which might complement the analyzed quantitative information. This underlines that an insufficient quality of documentation does not necessarily allow conclusions to be drawn about the quality of care, and vice versa.

Brown [31] emphasizes this by cautioning people to always consider the circumstances under which people put information into the record before drawing conclusions. This is a major issue because the completeness of documentation might be biased due to aspects that do not directly derive from clinical care. On the one hand, the hospital’s reimbursement for the delivered care depends on what is documented and might cause a possible strengthened thorough filling of certain fields [32]. On the other hand, the burden caused by documentation tasks is critically heavy. It is responsible for a high prevalence of burnout among physicians and nurses [33]. Therefore, clinically or legally unnecessary documentation might be evaded [34]. However, even though complete documentation might neither necessarily arise from nor be essential for the delivery of excellent clinical care, it is likewise of concern under the aspect of big data analytics. In this regard, it would be desirable for the discussed diagnosis to indeed be present in the EMR, even if it already exists in the HIS. An automatic transfer of this information could help to prevent the burden on staff resulting from manual transmission and ensure a complete data set. This is an important point, as the insights gained from analyzing big data offer numerous opportunities, like data-based personalized care in diagnostics and therapy or the support of scientific activities, both with the chance of saving lives and reducing health care costs at the same time [7,35]. It is therefore indispensable to recognize the possibility of changes in documentation due to the implementation or adaptation of EMRs. Only with this attention will it become possible to optimize the documentation process with a focus on the various benefits for all stakeholders, like patients [6], practitioners [36], organizations [5], and society [7].

### Strengths and Limitations

The German health care system, in which the study was conducted, was heavily strained by the high number of COVID-19 cases and the associated use of intensive care units during the study period. Especially the first measuring phase (November 2020) fell into the first pandemic year when many planned procedures were suspended to increase hospital capacities. For the first lockdown period in Germany (March 2020), a decrease in orthopedic surgeries is described by approximately 80% [37]. A lockdown-like situation was again declared during the first measuring phase [38], which probably explains the difference in treated patients over the 2 measuring phases (n_Paper_=44 vs n_Electronic_=136). However, the similarity between the coded ICD diagnoses over different years (Table 1) suggests that the proven changes in completeness of documentation are not due to significant changes in the studied patient sample, but a detailed sample description based on socioeconomical data is missing due to data protection regulations. On the other hand, there is a study assuming a positive influence of the pandemic on the completeness of documentation since an incomplete documentation might have led to repetitive contacts with the patient, which could have been avoided if the documentation would have been complete in the first place [39]. However, this cannot be verified in this paper due to the lack of further measuring phases. Within this given context, the generalizability of the presented results remains limited.

Further, limitations regarding the analyzed data set have to be stated. The chosen unpaired *t* test is theoretically based on the assumption of normal distributions. This could not be confirmed statistically for the mean completeness scores by the Shapiro-Wilk test. Although *t* test has been shown to be robust to a missing normal distribution [40] and the QQ plots (Multimedia Appendix 2) indicate an approximation to a normal distribution, the results could still be biased by the broken assumption.

Moreover, the analyzed data set is missing any information on which person was entering the documentation regarding which patient. On the one hand, it might be arguable that the same physicians or nurses were documenting during the first and also the second measuring phases. This circumstance would make the 2 compared measuring phases dependent samples, having an impact on the chosen statistical model. Since the analyzed data set is missing this information, the results might be biased regarding a possible dependent or independent sample. However, the time passed between the 2 measuring phases might have led to a change of the employees since the teaching status of the hospital results in many young physicians or nurses who do not necessarily stay on the same ward for a long time. Moreover, the hospital in which the study was conducted has a rotation system in which clinicians rotate hospital-wide across different wards of the same discipline. Those 2 facts let us assume that the 2 compared samples are indeed independent. However, the lack of information regarding the documenting individual is preventing the use of advanced tests like mixed effect models. These could equally consider the record type on the one hand and the possible documenting individuals on the other hand, potentially advancing the results’ reliability. However, the 15-month interval from the implementation date of the EMR to the second data collection signifies that there is only little risk of any possible changes in documentation due to a bias from the described effects of preimplementational documentation training [41] since the employees indeed underwent software training before they were allowed to use the EMR. Therefore, the shown changes in completeness are, in fact, most likely due to the implementation of the EMR.

### Conclusions

The results show that implementing EMRs can influence the completeness of documentation. A demonstrated improved completeness might also facilitate an improvement of the described outcomes that depend on documentation that is of high quality, like the availability [4] and analyzability of information [7,35], the coordination of care [5], or patient safety [6]. However, at the same time, the results show that a deterioration of completeness is also conceivable with the accompanied risks. This highlights the importance of understanding the underlying mechanisms that determine these changes. The knowledge may help stakeholders manage the implementation of new EMRs or the optimization of existing EMRs. Future research should address mechanisms that can improve documentation while simultaneously reducing the burden on practitioners caused by documentation tasks.

## Appendix

Multimedia Appendix 1 STROBE Checklist.

Multimedia Appendix 2 Q-Q-Plots.

## Abbreviations

EMR: electronic medical record
HIS: hospital information system
ICD: International Classification of Diseases
STROBE: Strengthening the Reporting of Observational Studies in Epidemiology

## Data Availability

The data sets generated and analyzed during this study are available from the corresponding author on reasonable request.
