# Supplementary material for: The Implementation of an Electronic Medical Record in a German Hospital and the Change in Completeness of Documentation: Longitudinal Document Analysis
Source: JMIR Med Inform. 2024 Jan 19;12:e47761. doi: 10.2196/47761 (PMC10837754; doi:10.2196/47761)

Q-Q-Diagramm von Summenscore über alle 10 Variablen hinweg mit einer Ausprägung von 0 - 10

von v_recordtype= Papier

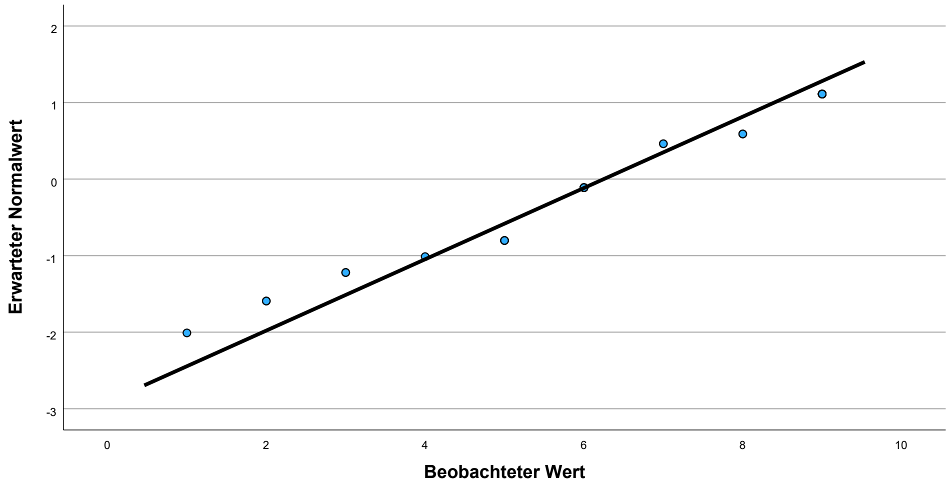

Q-Q-Diagramm von Summenscore über alle 10 Variablen hinweg mit einer Ausprägung von 0 - 10

von v_recordtype= Elektronisch

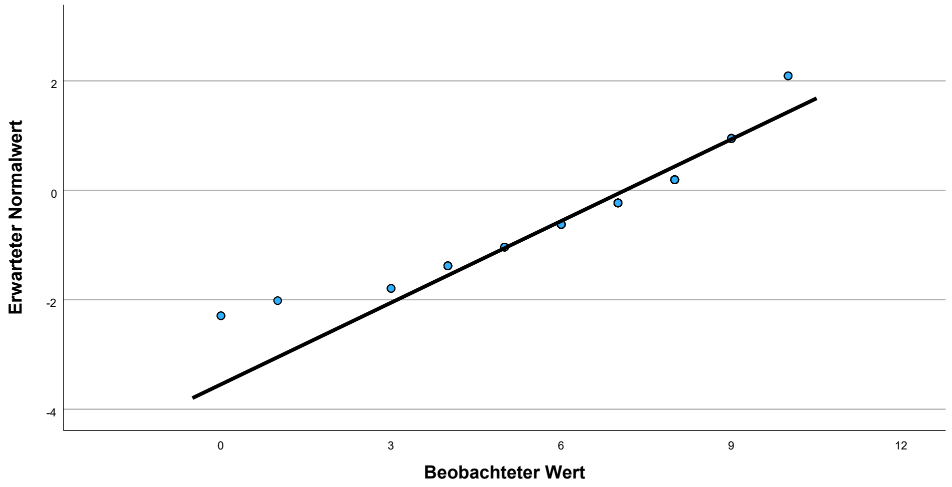

Supplement: Multimedia Appendix 2 [file medinform_v12i1e47761_app2.pdf]
